# Measuring health literacy for maternal health surveillance: psychometric validation of the Portuguese HLS_19_-Q12 among pregnant women

**DOI:** 10.3389/fpubh.2026.1920509

**Published:** 2026-08-28

**Authors:** Nuno Ferreira, Manuela Ferreira, Andreia Costa

**Affiliations:** 1 Nursing Research Innovation and Development Centre of Lisbon (CIDNUR), School of Nursing, Universidade de Lisboa, Lisbon, Portugal; 2 Unidade Local de Saúde Viseu Dão-Lafões, Viseu, Portugal; 3 Universidade Politécnica de Viseu, Escola Superior de Saúde, Viseu, Portugal; Univ Coimbra, UICISA:E, Coimbra, Portugal; 4 Instituto de Saúde Ambiental da Faculdade de Medicina da Universidade de Lisboa, Lisboa, Portugal; 5 Laboratório para a Sustentabilidade do Uso da Terra e dos Serviços dos Ecossistemas - TERRA, Lisboa, Portugal

**Keywords:** antenatal care, health literacy, HLS19-Q12, maternal health, Portugal, pregnant women, psychometric validation

## Abstract

Health literacy is a modifiable determinant of maternal health and an important condition for equitable access to antenatal information, shared decision-making, and preventive care. Reliable and brief instruments are needed to monitor health literacy during pregnancy and to guide public health interventions. This study validated the Portuguese version of the 12-item European Health Literacy Survey questionnaire (HLS19-Q12) among pregnant women and examined its psychometric properties among pregnant women. A descriptive cross-sectional psychometric study was conducted with 886 pregnant women recruited through antenatal care services in Portugal. Factorial validity was assessed using exploratory factor analysis and confirmatory factor analysis with the diagonally weighted least squares (DWLS) estimator; reliability was evaluated using Cronbach’s alpha and McDonald’s omega; and construct validity was examined through correlations with digital and navigational health literacy indices. The results supported a unidimensional structure for the HLS19-Q12 [χ^2^(50) = 221.77, *p* < 0.001; CFI = 0.993; TLI = 0.991; RMSEA = 0.062, 90% CI (0.054, 0.071); SRMR = 0.046]. Standardized factor loadings ranged from 0.59 to 0.80. Internal consistency was high (*α* = 0.86; *ω* = 0.87), and moderate positive correlations with digital (r = 0.65) and navigational (*r* = 0.61) health literacy supported convergent validity. The Portuguese HLS_19_-Q12 is a valid and reliable instrument for assessing general health literacy among pregnant women. Following further validation and implementation research, the Portuguese HLS_19_-Q12 may have potential to support population monitoring of general health literacy among pregnant women and to inform targeted public health interventions.

## Introduction

Health literacy (HL) is increasingly recognized as a public health capacity, not only as an individual attribute. It reflects the ability to access, understand, appraise, and apply health information in ways that support informed decisions about healthcare, disease prevention, and health promotion (1). Adequate HL contributes to self-management, participation in care, and appropriate use of services, whereas limited HL is consistently associated with delayed care, lower adherence to professional advice, avoidable morbidity, and health inequities (2). For public health systems, measuring HL is therefore essential for identifying informational barriers, monitoring inequities, and designing literacy-sensitive services.

Pregnancy is a critical window for health promotion and preventive intervention. Pregnant women must interpret and act on complex information about nutrition, medication, screening, warning signs, mental health, birth preparation, breastfeeding, and newborn care while navigating multiple health services (3–6). Limited HL during pregnancy has been associated with adverse maternal and neonatal outcomes, including gestational diabetes, preterm birth, low birth weight, poorer maternal mental health, and lower engagement in antenatal education (3, 6). Because pregnancy involves repeated contact with health services, antenatal care also offers a strategic opportunity to identify HL needs early and to adapt communication, education, and referral pathways.

International evidence shows that HL levels among pregnant women vary substantially according to educational attainment, socioeconomic position, language, migration background, and healthcare context (4, 5). Studies have reported that between 15 and 50% of pregnant women demonstrate inadequate or problematic HL (7). Women with lower income, younger age, lower educational attainment, or migrant backgrounds may experience additional barriers, including fragmented information, linguistic obstacles, limited digital access, and difficulty navigating services (7). These patterns highlight the need for brief, comparable measures that can identify population-level needs without increasing the burden of antenatal assessment.

In Portugal, HL has been identified as a determinant of healthcare access and utilization among pregnant women, influencing engagement with prenatal care and preventive health behaviors (8). Although the National Health Service provides universal coverage, disparities persist, particularly among women with lower educational attainment, migrant backgrounds, or more complex social circumstances. Strengthening HL during pregnancy is therefore closely aligned with maternal health equity, the quality of antenatal care, and the design of targeted public health programs.

To support international comparison and evidence-based policymaking, the WHO Action Network on Measuring Population and Organizational Health Literacy (M-POHL) developed a harmonized approach to population HL measurement (2). Within this framework, the European Health Literacy Survey developed a comprehensive conceptual model and corresponding instruments, including the short-form HLS_19_-Q12, which assesses HL across healthcare, disease prevention, and health promotion domains (2). Although the Portuguese version of the HLS_19_-Q12 has been validated in the general adult population (9), its psychometric performance among pregnant women has not yet been examined. This study aimed to evaluate the factorial structure, reliability, and construct validity of the Portuguese HLS_19_-Q12 among pregnant women and to explore its potential value as a public health tool for maternal health surveillance, service planning, and equity-oriented antenatal interventions.

## Materials and methods

### Instrument and scoring

General health literacy (HL) was assessed using the HLS_19_-Q12, a validated short-form instrument derived from the European Health Literacy Survey (HLS-EU-Q47) and developed within the WHO Action Network on Measuring Population and Organizational Health Literacy (M-POHL) framework (2). This scale is grounded in the conceptual model proposed by Sørensen et al., which defines HL as the ability to access, understand, appraise, and apply health-related information in everyday life across three domains: healthcare, disease prevention, and health promotion (1).

The Portuguese version of the HLS_19_-Q12 was previously validated in a representative national sample of adults aged 16 years and older, confirming its reliability and factorial validity for the general population (9). However, to the best of our knowledge, no psychometric validation of the HLS_19_-Q12 has previously been reported in a population of pregnant women.

The instrument comprises 12 items rated on a four-point Likert scale ranging from “very difficult” [1] to “very easy” [4]. Following the international scoring guidelines established by the HLS19 Consortium, individual item scores were standardized and transformed into a unified index ranging from 0 to 100, with higher values indicating greater HL. Respondents with fewer than 80% valid responses were excluded from computation.

The overall HL index, along with sub-indices reflecting the three domains and four information-processing dimensions (access, understand, appraise, and apply), was categorized into four proficiency levels: inadequate (0–50), problematic (50–66.67), sufficient (66.68–83.33), and excellent (>83.33).

The use of the HLS_19_-Q12 and the associated scoring framework in this study was authorized by the M-POHL Health Literacy Consortium (https://m-pohl.net/Design_Methods).

### Participants and procedures

Ethical approval for this study was obtained from the Institutional Health Ethics Committees of the Tondela-Viseu Hospital Centre (Ref. 10/29/09/2023) and the Central Regional Health Administration (Process No. 124-2023). All research procedures adhered to the ethical standards outlined in the Declaration of Helsinki (11). Written informed consent was obtained from all participants before enrolment.

Eligible participants were pregnant women aged 18 years or older, with a confirmed gestational age of at least 10 weeks and sufficient fluency in Portuguese to complete the questionnaire. No additional exclusion criteria were applied. Data collection took place between 15 October 2023 and 15 May 2024.

Recruitment followed a multimodal, non-probabilistic convenience sampling strategy designed to maximize accessibility and participation. Pregnant women were invited to participate during routine antenatal care, regardless of whether they attended the public hospital, primary healthcare centers, or private obstetric consultations, as many women received care in more than one setting. Before choosing the preferred mode of questionnaire completion, all women received an explanation of the study from a member of the research team, who confirmed eligibility, including sufficient Portuguese-language proficiency to understand the study information, provide informed consent, and complete the questionnaire. Participants could then choose among three complementary modes of questionnaire completion: (1) face-to-face interviews conducted in hospital and primary care facilities; (2) a digital version accessible via QR code or web link; and (3) a self-administered paper version distributed during antenatal appointments. Among those who received the paper version, the response rate reached 76%. Overall, 402 women (45.4%) participated through face-to-face interviews, 255 (28.8%) completed the online questionnaire, and 229 (25.8%) completed the paper questionnaire. To minimize duplicate participation, women accessing the online questionnaire were informed in the consent form that they should not complete the survey if they had already participated through another recruitment route. For score computation, cases with fewer than 80% valid responses were excluded from the corresponding scale calculation. Consequently, the number of participants varied slightly across subscale analyses according to the availability of valid responses.

Construct validity was further examined using the Portuguese versions of the optional HLS19 Digital Health Literacy (HLS_19_-DIGI) and Navigational Health Literacy (HLS_19_-NAV) modules. Both instruments belong to the HLS19 measurement framework, use the same four-point response scale (“very difficult” to “very easy”), and are scored according to the HLS19 Consortium methodology, with higher scores indicating better health literacy in the respective domains.

### Statistical analysis

Statistical analyses were conducted using IBM® SPSS® Statistics (version 29.0) (12), jamovi (version 2.6.44) (13), Stata Statistical Software (Release 18.0) (10), and R (version 4.6.1) (14). The confirmatory factor analysis was performed using the lavaan package (version 0.7–2). First, descriptive analyses were performed to calculate absolute frequencies (n), percentages (%), means (M), and standard deviations (SD).

The internal consistency of the HLS_19_-Q12 was examined through Cronbach’s alpha (*α*) and McDonald’s omega (*ω*), in order to address the limitations of α and provide a more robust estimate of reliability. Cronbach’s alpha was reported to enable comparison with the original version of the instrument, while omega was computed using jamovi software to provide a more robust estimate by relaxing the tau-equivalence assumption (15). Reference values for interpretation were as follows: >0.90 excellent, 0.80–0.89 good, 0.70–0.79 acceptable, 0.60–0.69 questionable, 0.50–0.59 poor, and <0.50 unacceptable (15, 16). Corrected item–total correlations were also inspected, with values >0.30 considered satisfactory.

To examine the dimensionality of the HLS_19_-Q12, an exploratory factor analysis was initially conducted using principal component extraction based on the Pearson correlation matrix. Sampling adequacy was evaluated using the Kaiser–Meyer–Olkin (KMO) measure and Bartlett’s test of sphericity. Factor retention was assessed using four complementary criteria: (i) Kaiser’s eigenvalue >1 rule, (ii) scree plot inspection, (iii) Horn’s parallel analysis, and (iv) the Minimum Average Partial (MAP) test. Parallel analysis and MAP were performed in Stata Statistical Software (Release 18.0) (17–19).

Because the HLS_19_-Q12 is an established instrument with a previously validated factorial structure, the exploratory factor analysis was performed only to examine whether the dimensionality observed in the original instrument was retained in this specific population of pregnant women. The subsequent confirmatory factor analysis was conducted to evaluate the fit of the hypothesized one-factor model in the same sample.

Subsequently, a Confirmatory Factor Analysis (CFA) was performed using the diagonally weighted least squares (DWLS) estimator implemented in the lavaan package (version 0.7–2) in R (version 4.6.1), which is recommended for ordinal Likert-type items (16, 20, 21). Model fit was evaluated using the chi-square statistic (χ^2^), Comparative Fit Index (CFI), Tucker–Lewis Index (TLI), Root Mean Square Error of Approximation (RMSEA, with 90% confidence interval), and Standardized Root Mean Square Residual (SRMR). The following cut-off criteria were adopted: CFI and TLI > 0.90, RMSEA < 0.08 (≤0.05 indicating excellent fit), and SRMR < 0.08 (16, 22, 23).

Construct validity was examined using Pearson correlation coefficients between the HLS_19_-Q12 total score and the HLS_19_-DIGI and HLS_19_-NAV total scores. For local fit evaluation, standardized factor loadings (*λ*) ≥ 0.50 and item reliability (R^2^) ≥ 0.25 were considered acceptable. Pearson’s correlation coefficients were used to examine associations with related constructs, with interpretation based on the following thresholds: <0.20 very low, 0.20–0.39 low, 0.40–0.69 moderate, 0.70–0.89 high, and ≥0.90 very high (16). Statistical significance was set at *p* < 0.05.

## Results

### Sample characteristics

The study included 886 pregnant women aged between 18 and 51 years (*M* = 31.09, SD = 5.60). Most participants resided in the Viseu district of Portugal (89.5%) and self-identified as Portuguese nationals (81.2%). Educational attainment was relatively high, with 42.6% holding a university degree and 40.7% having completed secondary education. The majority were married or cohabiting (73.4%), and 77.8% were professionally active.

The mean gestational age was 29.6 weeks, with most women in the third trimester (68.3%), followed by 12.6% in the second trimester and 19.1% in the first trimester. Approximately two-thirds of pregnancies were planned (66.6%), and 74.2% were classified as low risk. Preconception care was reported by 57.3% of participants, and 88.8% initiated antenatal care during the first trimester.

Regarding health status and behaviors, 19.2% reported a chronic condition or disability. One-third of participants reported tobacco smoke exposure (32.3%), and 33.2% reported alcohol or psychoactive substance use either before or during pregnancy. Physical activity levels varied, with one-quarter reporting inactivity. Pre-pregnancy BMI indicated that 24.5% were overweight and 15.0% obese. Most participants expressed an intention to breastfeed (92.6%).

### Descriptive statistics of the HLS_19_-Q12 items

Descriptive statistics for the HLS19-Q12 items are presented in Table 1. The items with the highest mean scores, reflecting greater perceived ease, were item 4 (“…to act on advice from your doctor or pharmacist?”; *M* = 3.27, SD = 0.47) and item 7 (“…to judge if information on unhealthy habits such as smoking, low physical activity, or drinking too much alcohol is reliable?”; *M* = 3.15, SD = 0.57). In contrast, the lowest mean scores were observed for item 5 (“…to find information on how to handle mental health problems?”; *M* = 2.62, SD = 0.75) and item 3 (“…to judge the advantages and disadvantages of different treatment options?”; *M* = 2.75, SD = 0.70), indicating greater difficulty in these domains.

**Table 1:** Descriptive statistics of the HLS_19_-Q12 items (*n* = 886).

Item	Mean	SD^1^	Skewness	Kurtosis
1. …to find out where to get professional help when you are ill?	2.96	0.63	−0.86	3.24
2. …to understand information about what to do in a medical emergency?	2.90	0.63	−1.61	6.14
3. …to judge the advantages and disadvantages of different treatment options?	2.75	0.70	−1.75	5.07
4. …to act on advice from your doctor or pharmacist?	3.27	0.47	0.34	1.50
5. …to find information on how to handle mental health problems?	2.62	0.75	−1.02	2.25
6. …to understand information about recommended health screenings or examinations?	2.97	0.61	−1.18	5.08
7. …to judge if information on unhealthy habits, such as smoking, low physical activity or drinking too much alcohol, are reliable?	3.15	0.57	−0.96	5.50
8. …to decide how you can protect yourself from illness using information from the mass media?	2.96	0.60	−1.88	8.44
9. …to find information on healthy lifestyles such as physical exercise, healthy food or nutrition?	3.14	0.51	−0.55	5.12
10. …to understand advice concerning your health from family or friends?	2.97	0.56	−1.94	9.86
11. …to judge how your housing conditions may affect your health and well-being?	3.08	0.51	−1.23	9.34
12. …to make decisions to improve your health and well-being?	3.00	0.50	−0.46	3.17

^1^SD, standard deviation.

Skewness values ranged from −1.94 to 0.34. Although some items showed approximately symmetric distributions, several demonstrated moderate to substantial negative skewness, indicating a concentration of responses in the higher response categories. Kurtosis values varied considerably, with some items showing marked leptokurtosis.

### Factorial structure of the HLS19-Q12

#### Exploratory factor analysis (EFA)

**Figure 1:**
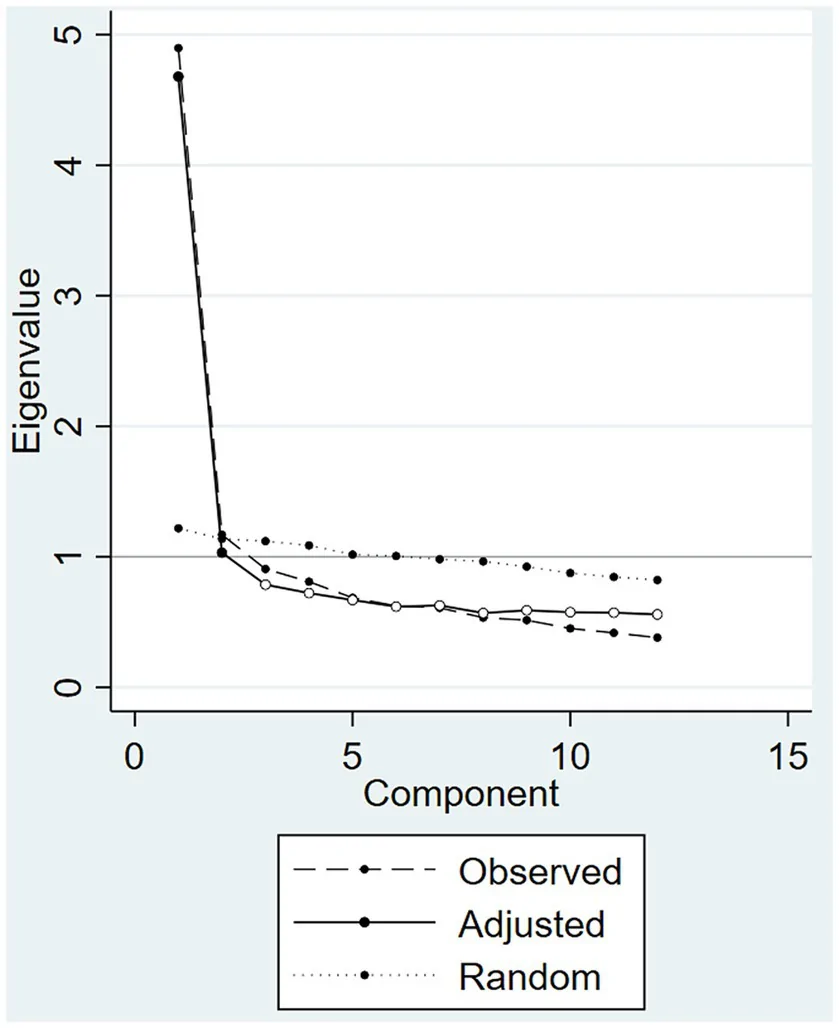
Scree plot and Horn’s parallel analysis of the HLS_19_-Q12, supporting the retention of a one-factor solution.

Sampling adequacy was confirmed (KMO = 0.902; Bartlett’s test of sphericity: χ^2^(66) = 3374.94, *p* < 0.001). The Kaiser criterion suggested a two-factor solution (eigenvalues = 4.897 and 1.169), with the first component accounting for 40.81% of the total variance. Visual inspection of the scree plot (Figure 1) indicated a clear inflection after the first component. Horn’s parallel analysis supported the retention of a single component when applying the more conservative 95th percentile criterion, and the Minimum Average Partial (MAP) procedure also supported a one-factor solution. Based on these complementary criteria and the theoretical unidimensional structure of the HLS_19_-Q12, a one-factor solution was retained for confirmatory factor analysis.

#### Confirmatory factor analysis (CFA)

**Figure 2:**
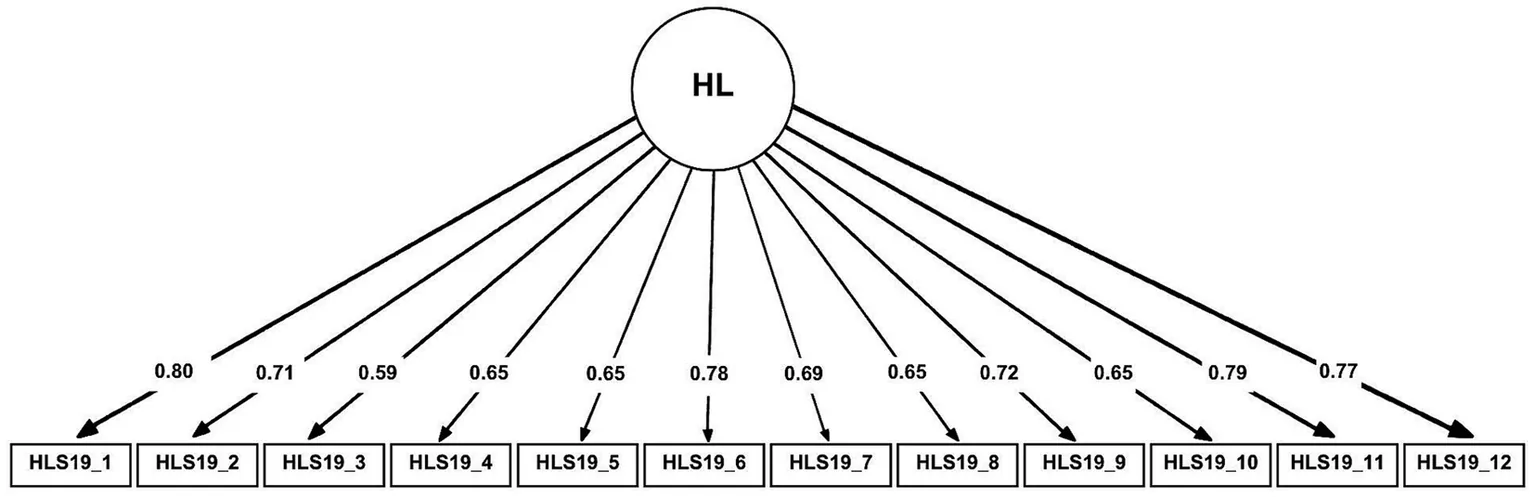
Standardized factor loadings for the one-factor confirmatory factor analysis (CFA) model of the Portuguese HLS_19_-Q12 estimated using the diagonally weighted least squares (DWLS) estimator.

The one-factor structure was tested using CFA with the diagonally weighted least squares (DWLS) estimator. The model demonstrated good overall fit: χ^2^(50) = 221.77, *p* < 0.001; CFI = 0.993; TLI = 0.991; RMSEA = 0.062, 90% CI [0.054, 0.071]; and SRMR = 0.046. These indices support the adequacy of the unidimensional model. Standardized factor loadings ranged from 0.59 (item 3) to 0.80 (item 1), with all items loading significantly on the latent construct (*p* < 0.001) (Figure 2). No correlated residuals or *post hoc* model modifications were specified.

### Construct validity and reliability

Evidence consistent with convergent validity was provided by moderate positive correlations between the HLS_19_-Q12 and the optional HLS_19_ modules: Digital Health Literacy (HLS19-DIGI; *r* = 0.65, *p* < 0.001) and Navigational Health Literacy (HLS_19_-NAV; *r* = 0.61, *p* < 0.001).

Internal consistency was high, with Cronbach’s alpha = 0.86 and McDonald’s omega = 0.87. Corrected item-total correlations ranged from 0.47 to 0.62, all above the recommended threshold of 0.30, confirming adequate homogeneity across items. Item reliability estimates (R^2^) ranged from approximately 0.34 to 0.64, indicating that the latent factor explained between 34 and 64% of the variance in the individual items. Reliability coefficients remained stable if any item was removed (*α* = 0.85–0.86; *ω* = 0.85–0.86), indicating robust internal consistency without reliance on any single item. Factor loadings from the confirmatory factor analysis and correlations with related constructs are presented in Table 2.

**Table 2:** Standardized factor loadings and correlations with related constructs.

HLS_19_	Factor loading
*On a scale from very difficult to very easy, how easy would you say it is…*	
1. …to find out where to get professional help when you are ill?	0.67
2. …to understand information about what to do in a medical emergency?	0.58
3. …to judge the advantages and disadvantages of different treatment options?	0.47
4. …to act on advice from your doctor or pharmacist?	0.54
5. …to find information on how to handle mental health problems?	0.56
6. …to understand information about recommended health screenings or examinations?	0.68
7. …to judge if information on unhealthy habits, such as smoking, low physical activity or drinking too much alcohol, are reliable?	0.60
8. …to decide how you can protect yourself from illness using information from the mass media?	0.52
9. …to find information on healthy lifestyles such as physical exercise, healthy food or nutrition?	0.64
10. …to understand advice concerning your health from family or friends?	0.49
11. …to judge how your housing conditions may affect your health and well-being?	0.63
12. …to make decisions to improve your health and well-being?	0.64

HL packages	Digital health literacy (HLS_19_-DIGI)	Navigation health literacy (HLS_19_-NAV)
Health literacy (HLS_19_)	0.65	0.61

All coefficients are statistically significant (*p* < 0.001).

The overall mean score for general health literacy was 68.31 (SD = 10.92). Across the six sub-indices, mean values were slightly higher, varying between 68.32 (SD = 16.77) for the Access dimension and 72.50 (SD = 11.30) for the Apply dimension of health information processing. The Disease Prevention domain showed a marginally lower mean of 67.48 (SD = 12.83), suggesting that the ability to locate and interpret preventive health information may represent a relatively greater challenge within this population.

## Discussion

This study provides evidence that the Portuguese version of the HLS_19_-Q12 is a valid and reliable instrument for assessing general health literacy among pregnant women. The results confirmed a unidimensional factorial structure, high internal consistency, and meaningful correlations with digital and navigational health literacy. These findings indicate that the instrument retains its conceptual coherence in a population with specific informational needs and frequent interaction with health services.

The unidimensional structure is consistent with the model proposed by the European Health Literacy Survey Consortium and with previous national validations in general adult populations (2, 9). The standardized factor loadings were generally moderate to high and comparable with those reported in the French, Spanish, and Chinese versions of the instrument (24–26). This supports the cross-cultural stability of the HLS_19_-Q12 and suggests that it can capture an overarching health literacy construct during pregnancy, a life-course period marked by specific clinical, emotional, and social demands.

The item-level pattern has practical relevance for maternal health services. Items related to understanding and acting on professional advice were easier for participants, whereas lower scores were observed for finding information on mental health problems and judging the advantages and disadvantages of treatment options. This suggests that pregnant women may feel more confident when following direct professional guidance but experience greater difficulty when appraising complex, uncertain, or preference-sensitive information. In antenatal care, literacy-sensitive communication should therefore go beyond simplifying instructions and should also support deliberation, critical appraisal of information, and shared decision-making.

The moderate positive correlations with digital and navigational health literacy reinforce the idea that general health literacy is connected to broader competencies required in contemporary health systems. Pregnant women increasingly access information through digital channels, appointment platforms, institutional websites, social media, and online communities, while also navigating referrals, screening schedules, primary care, hospital-based care, and public or private service pathways. The HLS_19_-Q12 may therefore be interpreted not only as a psychometric scale but also as a short population indicator of general health literacy among pregnant women within increasingly complex and digitalized maternal healthcare systems.

### Public health implications

The following implications should be interpreted as potential applications requiring confirmation through further validation and implementation research. Subject to further validation, the HLS_19_-Q12 may have potential as a brief assessment tool in antenatal care to identify health literacy-related barriers that may affect communication, informed consent, adherence to recommendations, and participation in health promotion activities. Used appropriately, the instrument should not be understood as labelling individual women as deficient, but as helping professionals adapt communication, provide clearer materials, and connect women with additional support.

At population level, routine or periodic use of the HLS_19_-Q12 could support regional monitoring of general health literacy among pregnant women. Aggregated data may help local health authorities identify geographic, social, or service-level patterns in literacy needs, particularly in areas with higher proportions of migrant women, lower educational attainment, or reduced access to antenatal education. Such monitoring could complement existing maternal health indicators by adding information on women’s ability to access, understand, appraise, and use health information.

The findings also point to priority areas for intervention. Greater difficulty in mental health information seeking and treatment appraisal suggests the need for antenatal education and digital resources that strengthen critical appraisal of information, service navigation, preparation for shared decision-making, and access to reliable mental health support during pregnancy. Integrating health literacy measurement into maternal health surveillance may therefore contribute to proportionate universalism: maintaining universal antenatal services while intensifying support for groups facing greater informational and navigational barriers.

The HLS_19_-Q12 may support the development of organizational health literacy within maternity services. Assessing health literacy should not be viewed merely as a means of identifying individual deficits, but rather as an opportunity for healthcare organizations to become more health literacy-responsive by adapting communication, simplifying health information, strengthening navigation support, and promoting shared decision-making. At the organizational level, aggregated HLS_19_-Q12 data may help maternity services identify population needs, inform staff training, evaluate literacy-sensitive interventions, and support continuous quality improvement within antenatal care. This perspective is consistent with current WHO recommendations emphasizing organizational responsiveness as a key component of health literacy development (27).

### Strengths and limitations

A strength of this study is the large sample of pregnant women and the use of a multimodal data collection strategy combining online, interviewer-administered, and self-completed paper formats. This approach likely improved accessibility for women with different educational and digital backgrounds. Although different administration modes may influence response patterns, identical questionnaire wording, response options, and scoring procedures were used across all formats. Formal analyses of administration-mode effects were beyond the scope of the present study and should be explored in future research. The use of internationally standardized scoring procedures and multiple indicators of validity and reliability also strengthens comparability with other HLS19 studies. Although the HLS_19_-Q12 has been previously validated, the use of the same sample for both exploratory and confirmatory factor analyses may have resulted in optimistic estimates of model fit. Independent cross-validation in future studies would further strengthen the evidence for the factorial stability of the instrument in pregnant populations.

Several limitations should be considered. The cross-sectional design precludes conclusions about temporal stability or changes in health literacy across pregnancy. Although the sample represented a substantial proportion of pregnancies in the Viseu district during the study period, it was not nationally representative and did not include stratification or weighting procedures. Further national validation is needed to confirm the instrument’s performance across Portuguese regions and different models of antenatal care. Measurement invariance should also be tested across Portuguese and migrant women, educational groups, age groups, parity, and care settings. In addition, future studies should examine the feasibility of applying the HLS_19_-Q12 in primary healthcare, hospital antenatal clinics, and community-based programs, as well as its usefulness for routine epidemiological surveillance. Self-reported data may also be affected by social desirability or perceived ease rather than observed performance; future research could therefore combine health literacy measurement with qualitative or implementation studies. The correlations with the HLS_19_-DIGI and HLS_19_-NAV modules provide supportive evidence of convergent validity. However, because these measures derive from the same conceptual framework and use similar response formats, they should not be considered fully independent validation criteria. Future studies should examine associations with external measures of health-related knowledge, decision-making, healthcare use, and maternal health behaviors.

### Future research and policy

Future studies should assess whether HLS_19_-Q12 scores predict antenatal care engagement, participation in childbirth preparation, uptake of preventive behaviors, shared decision-making, and maternal or neonatal outcomes. Longitudinal designs could clarify how health literacy changes across trimesters and postpartum, particularly as informational needs shift from pregnancy monitoring to birth, breastfeeding, and newborn care. At policy level, integrating general health literacy indicators into local and national monitoring systems could help align antenatal care with public health goals related to equity, universal health coverage, and person-centered maternity care.

## Conclusion

This study provides initial evidence of the structural validity and internal consistency of the Portuguese HLS_19_-Q12 among pregnant women, supporting its potential as a brief measure of general health literacy in the context of pregnancy. The unidimensional factorial structure, satisfactory reliability indices, and moderate associations with digital and navigational HL indicate that the instrument retains its conceptual integrity in a population with high informational and service-navigation demands.

Beyond its psychometric performance, the HLS_19_-Q12 offers practical value for public health. Following further evaluation of measurement invariance, temporal stability, predictive validity, responsiveness, and implementation feasibility, support population monitoring of general health literacy among pregnant women, help identify priority areas for antenatal education, and inform, and inform literacy-sensitive communication and equity-oriented interventions.

## Data Availability

The raw data supporting the conclusions of this article will be made available by the authors, without undue reservation.
